# SURVIVAL OF THE FITTEST OR FRAILEST? COMPARING THE HEALTH OF CENTENARIANS OVER A 10-YEAR PERIOD

**DOI:** 10.1093/geroni/igac059.2730

**Published:** 2022-12-20

**Authors:** Lian Ying Chun Pat, Bobo Hi Po Lau, Karen Siu Lan Cheung, Grace Man Yee Chan, Joseph Shiu Kwong Kwan, James Ka Hay Luk, Peter Martin, Cecilia Lai Wan Chan

**Affiliations:** Hong Kong Shue Yan University, Hong Kong, Hong Kong; Hong Kong Shue Yan University, Hong Kong, Hong Kong; The University of Hong Kong, Hong Kong, Hong Kong; The Hong Kong Council of Service, Hong Kong, Hong Kong; Imperial College London, London, England, United Kingdom; TWGHs Fung Yiu King Hospital (FYKH), Hospital Authority, Hong Kong, Hong Kong; Iowa State University, Ames, Iowa, United States; The University of Hong Kong, Hong Kong, Hong Kong

## Abstract

The first centenarian study in Hong Kong was conducted in 2011 and examined the multidimensional health of adults aged 95 or older. The 2011 study found that, among a population of about 3,000 centenarians, a significant proportion enjoyed a high degree of autonomy in their daily functions in relatively good health. The study has been repeated in 2021/22 (i.e., born in 1926 or before) when the city had more than 11,000 centenarians. Comparison of the two samples (2011: Nf77; 2021/22: Nf120) who lived with their family shows a significant difference in functional health, but not as much for physical health, favouring the 2011 cohort. More than 75% of the 2011 cohort demonstrated autonomy in activities of daily living (Bathing: 77.9%, dressing: 85.7%, toileting: 90.9%, indoor transfer: 89.6%; continence:75.3% and feeding: 94.8%). Only about half of the 2021/22 cohort were autonomous in these areas (40.0%, 44.3%, 54.7%, 42.5%, 63.2%, 46.7%, respectively). The number of chronic illnesses between the two cohorts were comparable (Mean(SD): 2011: 2.7 (1.6); 2021: 3.26 (1.60), yet dementia and frailty were more prevalent in the 2021 cohort (dementia: 44%; frailty: 9.1%) then the 2011 cohort (41.0%; 23.4%). Our findings alert metropolitans worldwide to the fast-increasing population of adults of advanced age with significant personal care and health needs in the community. Existing care for older adults has to be reframed and overhauled to provide comprehensive home- and personal-care support which will be essential for realizing ageing-in-place for adults in advanced age, especially after social distancing policies in COVID-19.

